# Regional Disparities in Stroke Hospitalization and Mortality Associated with Long-Term Fine Particulate Matter Exposure: A Nationwide Ecological Study in Spain

**DOI:** 10.3390/medsci14040475

**Published:** 2026-08-12

**Authors:** José María Ramírez-Moreno, Víctor Martín Hernández, Andrea Parejo Olivera, Noelia Valverde Mata, Marina Mesa Hernández, Pablo Macías Sedas, Ana Maria Roa Montero, María José Gómez Baquero, David Ceberino Muñoz, David Tena Mora

**Affiliations:** 1 Stroke Center, Neurology Department, University Hospital of Badajoz, 06080 Badajoz, Extremadura, Spainanamaria.roa@salud-juntaex.es (A.M.R.M.);; 2 Department of Biomedical Sciences, University of Extremadura, 06080 Badajoz, Extremadura, Spain; 3 Pneumology Department, University Hospital of Badajoz, 06080 Badajoz, Extremadura, Spain; victormanuel.martin@salud-juntaex.es

**Keywords:** air pollution, stroke, hospitalization, particulate matter, PM2.5, ecological study, Spain, regional disparities, sex differences, mortality

## Abstract

Background/Objectives: Stroke remains a leading cause of death and disability globally, with considerable regional heterogeneity in incidence and mortality reflecting differences in classical vascular risk factors and environmental determinants. Fine particulate matter (PM2.5) is an established modifiable environmental risk factor, yet its association with regional stroke disparities remains understudied in Mediterranean European contexts. We examined regional differences in stroke hospitalization and mortality and their ecological associations with long-term air-pollution exposure across Spain’s 17 autonomous communities from 2013 to 2021. Methods: We conducted a nationwide longitudinal ecological study using aggregated stroke indicators from INCLASNS and ambient air-pollution data from MITECO. The dataset comprised 306 sex-specific region-year observations. The primary outcome was the age-standardized hospitalization rate (ASHR); secondary outcomes were CHR, CMR, ASMR, CFR, and IMI. Associations were assessed using correlation analyses, temporal trends, regional comparisons, and multivariable regression. Sex-stratified analyses were considered exploratory because pollutant concentrations were measured at the regional-year level and were not sex-specific. Results: Mean ASHR was 20.1 ± 5.4 per 10,000 population, with marked regional variation. PM2.5 showed the largest positive ecological correlation with ASMR among all pollutants examined (r = 0.264, *p* < 0.001), although the correlation remained weak. Overall pollutant–outcome correlations were weak and heterogeneous in direction. Regional variation in stroke burden was not adequately explained by the available air-pollution indicators. Conclusions: PM2.5 was weakly associated with ASMR, whereas associations with hospitalization and other pollutants were small and inconsistent. These ecological findings do not support causal or individual-level inference and warrant confirmation using higher-resolution exposure and individual-level data.

## 1. Introduction

Stroke remains the second leading cause of death globally and a major contributor to disability-adjusted life years [1]. In 2019, 12.2 million people experienced a first-ever stroke and 6.6 million deaths were attributed to cerebrovascular disease [2]. Considerable geographic heterogeneity in stroke incidence and mortality has been documented, reflecting variation in vascular risk factors, socioeconomic determinants, and healthcare infrastructure. Environmental exposures, particularly ambient air pollution, may also contribute to geographical differences in cerebrovascular burden [3].

Among these exposures, fine particulate matter ≤2.5 μm (PM2.5) is a well-established environmental risk factor associated with vascular morbidity and mortality. More than 90% of the global population is exposed to annual levels above the current WHO guideline of 5 μg/m^3^ [1]. Meta-analyses of over 90 cohort studies report modest but significant increases in stroke risk with long-term PM2.5 exposure, and the ESCAPE European cohort reported a hazard ratio of 1.19 per 5 μg/m^3^ increase [4], although with a confidence interval that did not exclude the null value [5]. Nevertheless, substantial heterogeneity in PM2.5–stroke associations persists across regions, influenced by differences in particle composition, atmospheric conditions, population susceptibility, and co-exposure patterns [6].

Evidence from Mediterranean Europe remains comparatively limited. Much of the available evidence originates from Asia, North America, and Northern Europe, where pollutant mixtures, meteorological conditions, and population characteristics may differ from those of Southern Europe [7,8]. Mediterranean regions are characterized by specific atmospheric processes, including seasonal thermal inversions, intense photochemical activity, and Saharan dust intrusions, which may influence particulate composition and regional exposure patterns. In Spain, nationwide longitudinal analyses integrating routinely collected cerebrovascular indicators with regional air-quality data remain scarce.

The availability of standardized health indicators and an extensive air-quality monitoring network provides an opportunity to examine whether annual regional pollutant concentrations are associated with, and help explain, geographical differences in stroke burden. However, analyses based on aggregated regional data are susceptible to ecological bias, exposure misclassification, and residual confounding and cannot establish individual-level or causal associations.

Given this context, the present study aimed to describe regional disparities in cerebrovascular hospitalization and mortality and examine their ecological associations with long-term regional PM2.5 concentrations across the 17 Spanish autonomous communities from 2013 to 2021. Secondary objectives included describing temporal patterns in health and air-quality indicators, exploring associations with other ambient pollutants, and conducting descriptive sex-stratified analyses.

## 2. Materials and Methods

### 2.1. Study Design and Setting

We conducted a nationwide longitudinal ecological study using aggregated community-level data from Spain’s 17 autonomous communities over nine years (2013–2021). The dataset included 306 sex-specific community-year outcome observations, derived from 17 autonomous communities, 9 calendar years, and 2 sex categories, covering approximately 47 million residents. These observations corresponded to 153 unique community-year environmental exposure units.

Stroke-related health indicators were available separately for men and women, whereas ambient air pollutant concentrations were measured at the community-year level and were not sex-specific. Consequently, male and female observations within the same community and year shared the same environmental exposure estimate and were not considered fully independent in pooled analyses. Analyses involving environmental exposures were interpreted as population-level ecological associations, and sex-stratified analyses were considered descriptive, exploratory, and hypothesis-generating.

This ecological design allowed the identification of regional disparities, describing temporal trends, and generating hypotheses for subsequent individual-level investigations, but it could not support individual-level causal inference. Regional co-occurrence of higher pollutant concentrations and higher stroke burden does not imply that the same individuals experienced both the highest exposure and the outcome; ecological fallacy and residual confounding therefore remain possible.

The study was reported in accordance with the Strengthening the Reporting of Observational Studies in Epidemiology (STROBE) guidelines, with the checklist provided in the Supplementary File.

### 2.2. Data Sources

Stroke-related health indicators were retrieved as aggregated, pre-calculated measures from the Key Indicators of the Spanish National Health System database (INCLASNS), maintained by the Spanish Ministry of Health. Data were available by autonomous community, sex, and calendar year for the period 2013–2021. The definitions, numerators, denominators, diagnostic codes, units, standardization procedures, and primary data sources were those specified in the corresponding INCLASNS indicator documentation sheets.

The crude hospitalization rate (CHR) and the age-standardized hospitalization rate (ASHR) were based on the annual number of patients discharged with a principal diagnosis of cerebrovascular disease, divided by the corresponding annual population and expressed per 10,000 population. Hospital discharges were identified using ICD-9-CM codes 430–438 through 2015 and ICD-10-ES codes I60–I69 from 2016 onwards, with both classifications mapped to category 0908 of the International Shortlist for Hospital Morbidity Tabulation. ASHR was standardized using the 2013 Eurostat European Standard Population. The underlying source for both hospitalization indicators was the Registry of Specialized Healthcare Activity–Minimum Basic Data Set (RAE-CMBD).

The age-standardized mortality rate (ASMR) included all deaths attributed to cerebrovascular disease (ICD-10 codes I60–I69) and was expressed per 100,000 population. CMR represented the age-standardized premature cerebrovascular mortality rate among persons younger than 75 years and was also expressed per 100,000 population. Both mortality indicators were derived from the Spanish National Statistics Institute cause-of-death and population statistics and were standardized using the 2013 Eurostat European Standard Population. CFR and IMI were retained as secondary hospital-based outcome indicators according to their official INCLASNS definitions.

### 2.3. Environmental Data

Ambient air-pollution data were obtained from the official historical air-quality datasets of the Spanish Ministry for the Ecological Transition and the Demographic Challenge (MITECO). These datasets integrate measurements from air-quality monitoring stations throughout Spain and provide geographical and technical metadata for each station.

Data were extracted for PM2.5, PM10, NO_2_, SO_2_, O_3_, CO, benzene (C_6_H_6_), and lead (Pb) for each year from 2013 to 2021. Monitoring stations were assigned to their corresponding autonomous community. For each pollutant and calendar year, the regional exposure value was calculated as the unweighted arithmetic mean of the available station-level annual means within each autonomous community. No geostatistical interpolation or population weighting was performed.

The composite air quality index (ICAe) was an exploratory composite pollutant concentration score calculated as ICAe = PM10 + O_3_ + SO_2_ + NO_2_. It was not equivalent to the official Spanish Air Quality Index and did not incorporate pollutant-specific thresholds or toxicological weights. Higher values represented only a larger numerical sum of the four component concentrations.

### 2.4. Study Population and Variables

Primary outcome: age-standardized hospitalization rate (ASHR). Secondary outcomes: CHR, CMR, ASMR, CFR, and IMI. Primary exposures were annual regional mean concentrations of PM2.5, PM10, NO_2_, SO_2_, O_3_, CO, benzene, and Pb. ICAe was not included in multipollutant regression because it was mathematically determined by four of the individual pollutant variables. Grouping variables included sex, calendar year, and autonomous community.

### 2.5. Statistical Analysis

Descriptive statistics were calculated for stroke-related health indicators and ambient air pollutant concentrations. Continuous variables were presented as mean ± standard deviation, median with interquartile range, and range, as appropriate.

Pearson correlation coefficients were used to describe crude ecological associations between pollutant concentrations and stroke-related health indicators. A total of 54 pollutant–health indicator combinations were examined. Nominal statistical significance was defined as *p* < 0.05, and a Bonferroni-corrected threshold of *p* < 0.0009 was additionally applied to account for multiple comparisons. Correlations not meeting the Bonferroni-corrected threshold were interpreted as exploratory.

Correlation matrices were stratified by sex. Because ambient pollutant concentrations were measured at the regional-year level and were not sex-specific, sex-stratified analyses were interpreted as descriptive and exploratory; no formal comparisons between sex-specific coefficients were performed.

Temporal trends were estimated using linear regression models with calendar year as a continuous variable, autonomous community as a fixed effect, and standard errors clustered by autonomous community. Models for health indicators additionally included sex. Regional variation was summarized descriptively using community-specific estimates rather than unadjusted one-way ANOVA and post hoc comparisons.

Exploratory linear regression models were constructed to evaluate associations between standardized pollutant concentrations and age-standardized stroke outcomes, including age-standardized hospitalization rate (ASHR) and age-standardized mortality rate (ASMR). Models were fitted sequentially as univariate pollutant models, models adjusted for calendar year, and multivariable models including co-pollutants and calendar year. Pollutant concentrations were mean-centered and scaled to one standard deviation. Regression coefficients therefore represent the change in the outcome associated with a one-standard-deviation increase in pollutant concentration.

Multivariable models included selected co-pollutants and calendar year. ICAe was not entered into multipollutant models because it was derived from several of the individual pollutants.

Multicollinearity among pollutants was assessed using variance inflation factors.

All statistical analyses were conducted using Python v3.9 with pandas, numpy, scipy, seaborn, and stats models. All *p*-values were two-tailed. For analyses other than the predefined correlation screening, *p* < 0.05 was considered nominally statistically significant.

### 2.6. Ethical Considerations

Only aggregated and anonymized regional data were used, with no individual-level identifiers. In accordance with Spanish Law 14/2007 on Biomedical Research, formal ethics committee approval was not required for studies that used public administrative datasets. Nevertheless, the protocol was registered with the institutional ethics committee. Data handling complied with GDPR (EU 2016/679) and Spanish Organic Law 3/2018. Data were stored on secure institutional servers adhering to FAIR principles.

## 3. Results

### 3.1. Descriptive Statistics

The final dataset comprised 306 sex-specific community-year observations from 17 autonomous communities over nine years (2013–2021) including 153 observations for men and 153 for women. These corresponded to 153 unique community-year environmental exposure units. Summary descriptive metrics for stroke-related health indicators and air pollutants are presented in Table 1.

Mean age-standardized hospitalization rate (ASHR) was 20.1 ± 5.4 per 10,000 population and crude hospitalization rate (CHR) 21.4 ± 4.6 per 10,000 population. Age-standardized mortality rate (ASMR) averaged 51.8 ± 11.3 per 100,000 population, and in-hospital mortality (CFR) was 29.5 ± 5.0%. Mean PM2.5 was 9.96 ± 1.88 μg/m^3^, PM10 19.3 ± 4.2 μg/m^3^, NO_2_ 15.0 ± 5.7 μg/m^3^. The composite air quality index (ICAe) averaged 48.0 ± 13.5.

### 3.2. Correlation Analysis

**Figure 1:**
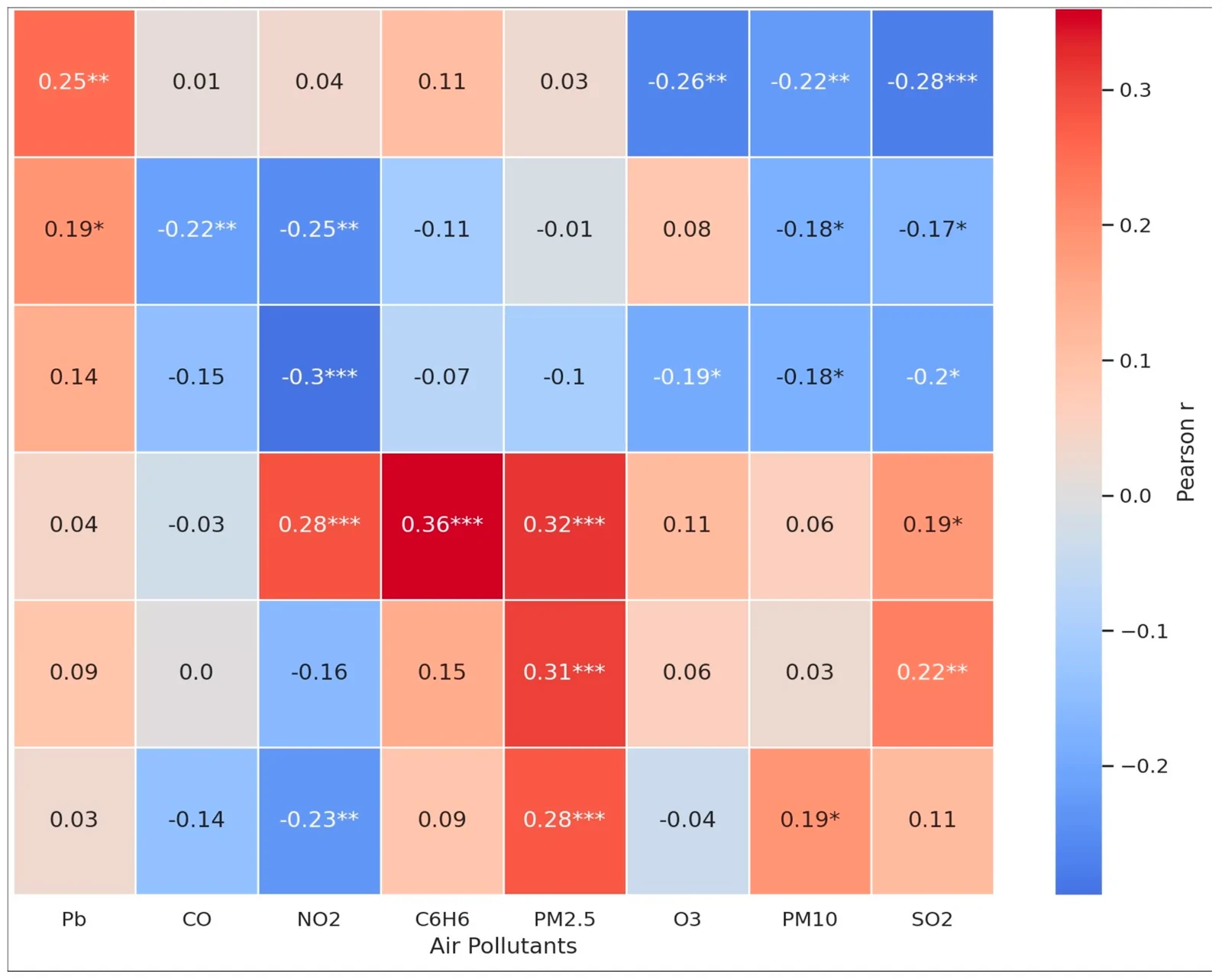
Correlation Heatmap Between Annual Regional Air-Pollution Measures and Cerebrovascular Health Indicators. Hierarchical clustering heatmap showing Pearson correlation coefficients (r) between eight ambient air pollutants and six stroke-related health indicators across 17 Spanish autonomous communities from 2013 to 2021. Red cells indicate positive correlations, and blue cells indicate inverse correlations. Asterisks denote statistical significance (* *p* < 0.05; ** *p* < 0.01; *** *p* < 0.001).

A total of 54 pollutant–health indicator combinations were examined, of which 23 (42.6%) reached nominal statistical significance at *p* < 0.05. However, only associations with *p* < 0.0009 met the Bonferroni-corrected threshold for multiple comparisons. Associations that did not meet this threshold were considered exploratory. Selected correlations with the largest absolute coefficients are presented in Table 2, and a visual summary of correlation strength and direction is provided in Figure 1.

PM2.5 demonstrated the largest positive correlation with age-standardized mortality rate (ASMR) (r = 0.264, *p* < 0.001). Benzene showed nominally significant positive correlations with ASHR (r = 0.185, *p* = 0.001) and ASMR (r = 0.128, *p* = 0.025).

Conversely, SO_2_, O_3_, and PM10 exhibited inverse correlations with hospitalization indicators: SO_2_ with crude hospitalization (r = −0.235, *p* < 0.001), O_3_ (r = −0.232, *p* < 0.001), and the Air Quality Index (r = −0.229, *p* < 0.001). In-hospital mortality (CFR) also showed inverse associations with Air Quality Index, NO_2_, and SO_2_.

**Figure 2:**
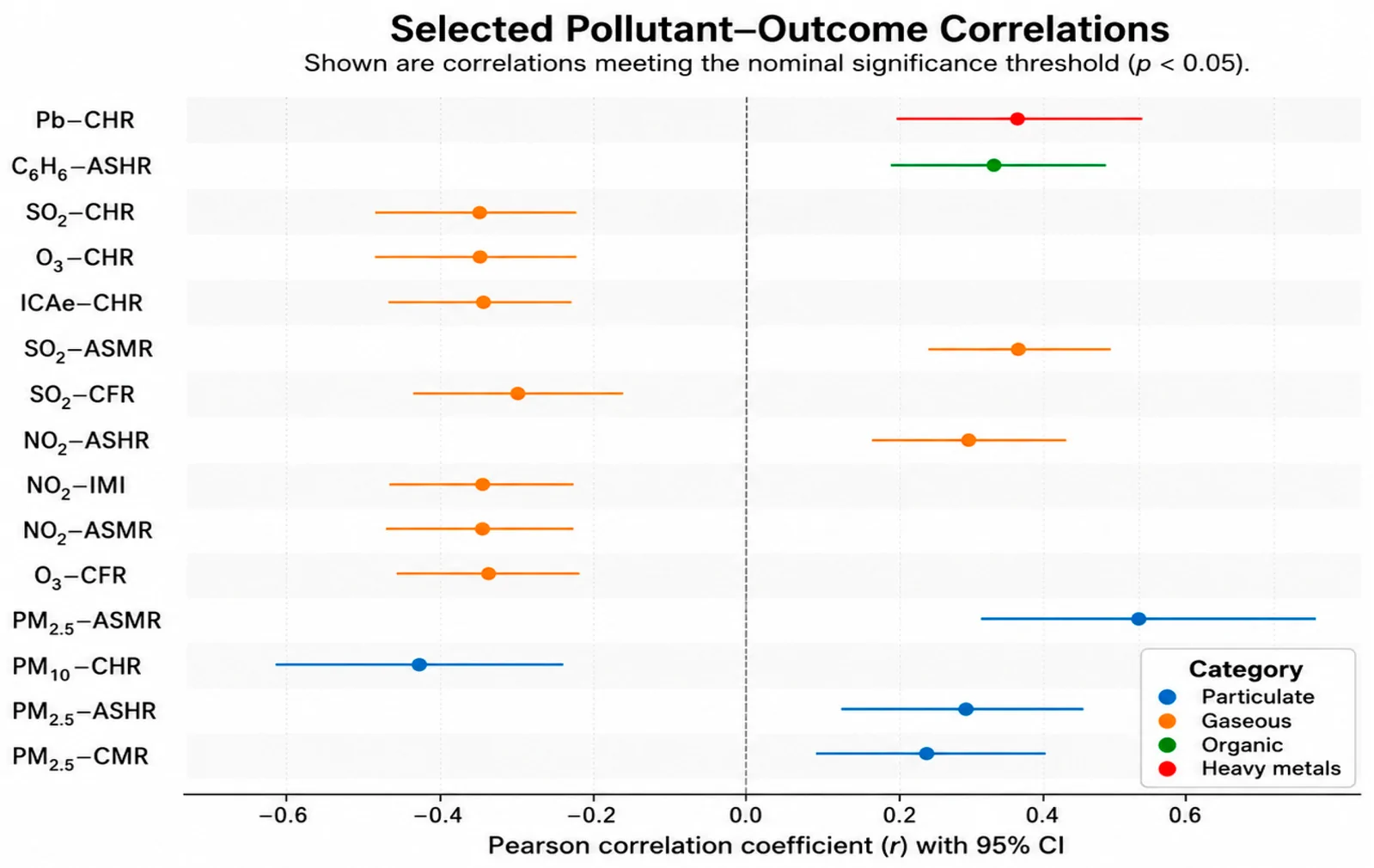
Forest Plot of Selected Correlations Between Annual Regional Air-Pollution Measures and Cerebrovascular Health Indicators. Forest plot of Pearson correlation coefficients (r) and 95% confidence intervals for pollutant–stroke outcome associations reaching nominal statistical significance at *p* < 0.05. Positive associations are displayed to the right of the null line and inverse associations to the left.

All correlations were weak in magnitude (|r| < 0.3), and no strong correlations were identified. These findings indicate limited explanatory relationships between annual regional pollutant concentrations and cerebrovascular health indicators. A forest plot summarizing the correlation coefficients is shown in Figure 2.

### 3.3. Sex-Stratified Analysis

**Figure 3:**
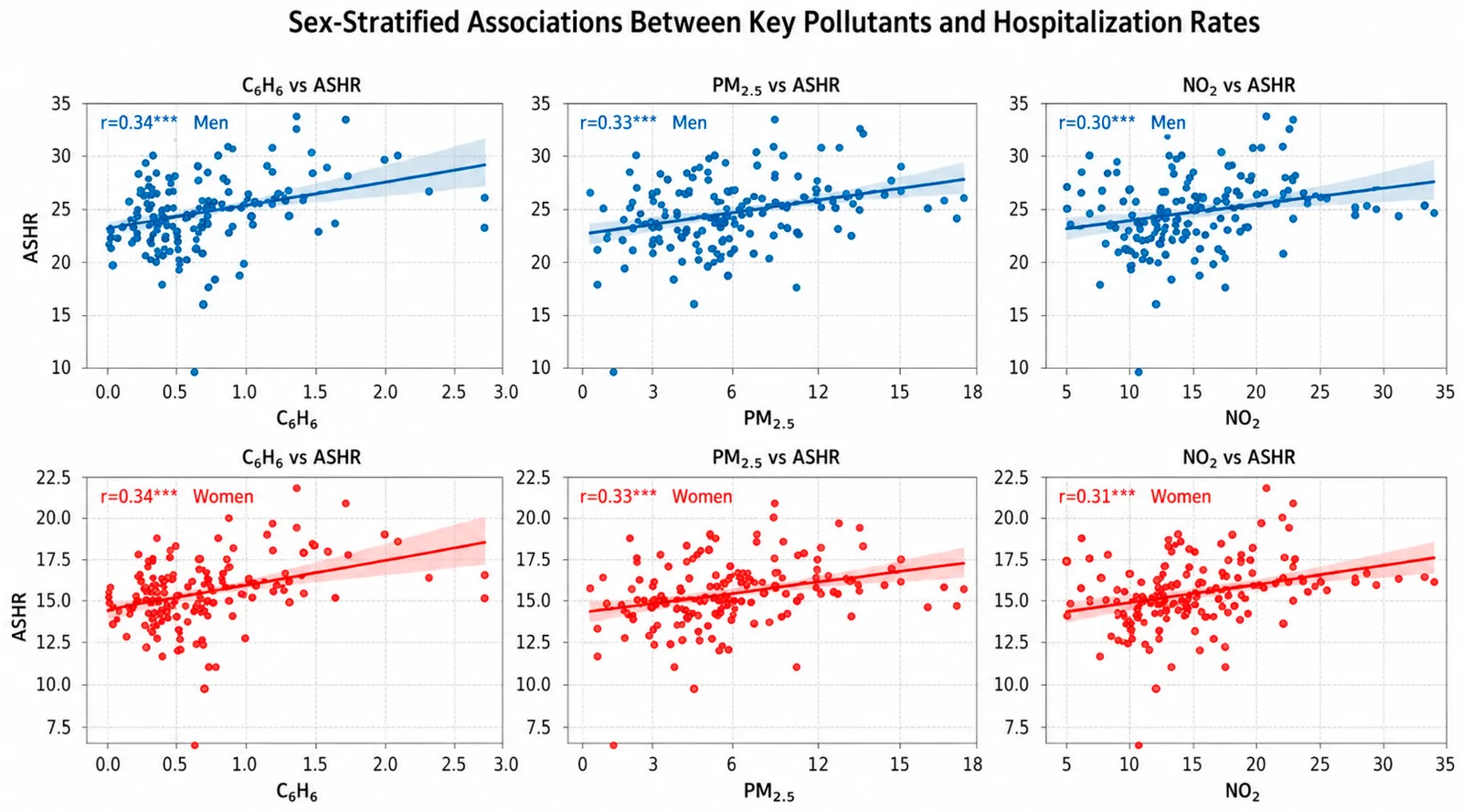
Sex-Stratified Associations Between Key Air Pollutants and Age-Standardized Stroke Hospitalization Rates. Scatter plots showing associations of benzene (C_6_H_6_), PM2.5, and NO_2_ with age-standardized stroke hospitalization rates, stratified by sex. Blue data points and regression lines correspond to men, whereas red data points and regression lines correspond to women. Each point represents a community-year observation. Regression lines and 95% confidence intervals are shown. Correlation coefficients are Pearson r values; *** indicate (*p* < 0.001).

Sex-stratified correlations are reported in Table 3, with a visual representation in Figure 3. Because men and women within the same autonomous community and year shared the same pollutant exposure estimate, these analyses were considered descriptive and exploratory.

Among men (*n* = 153): Benzene showed largest positive correlation with ASHR (r = 0.338, *p* < 0.001), followed by PM2.5 (r = 0.320, *p* < 0.001) and NO_2_ (r = 0.260, *p* = 0.001). Ozone demonstrated significant inverse correlations with CHR (r = −0.268, *p* < 0.001).

Among women (*n* = 153): Benzene exhibited even stronger association (r = 0.364, *p* < 0.001). NO_2_ (r = 0.308, *p* < 0.001) and PM2.5 (r = 0.296, *p* < 0.001) showed modest positive correlations, whereas SO_2_ showed an inverse correlation with CHR (r = −0.312, *p* < 0.001).

Although some coefficients differed numerically between men and women, no formal between-sex comparisons were performed because exposure estimates were not sex-specific and the sex-stratified observations were not independent.

### 3.4. Temporal Trends

**Figure 4:**
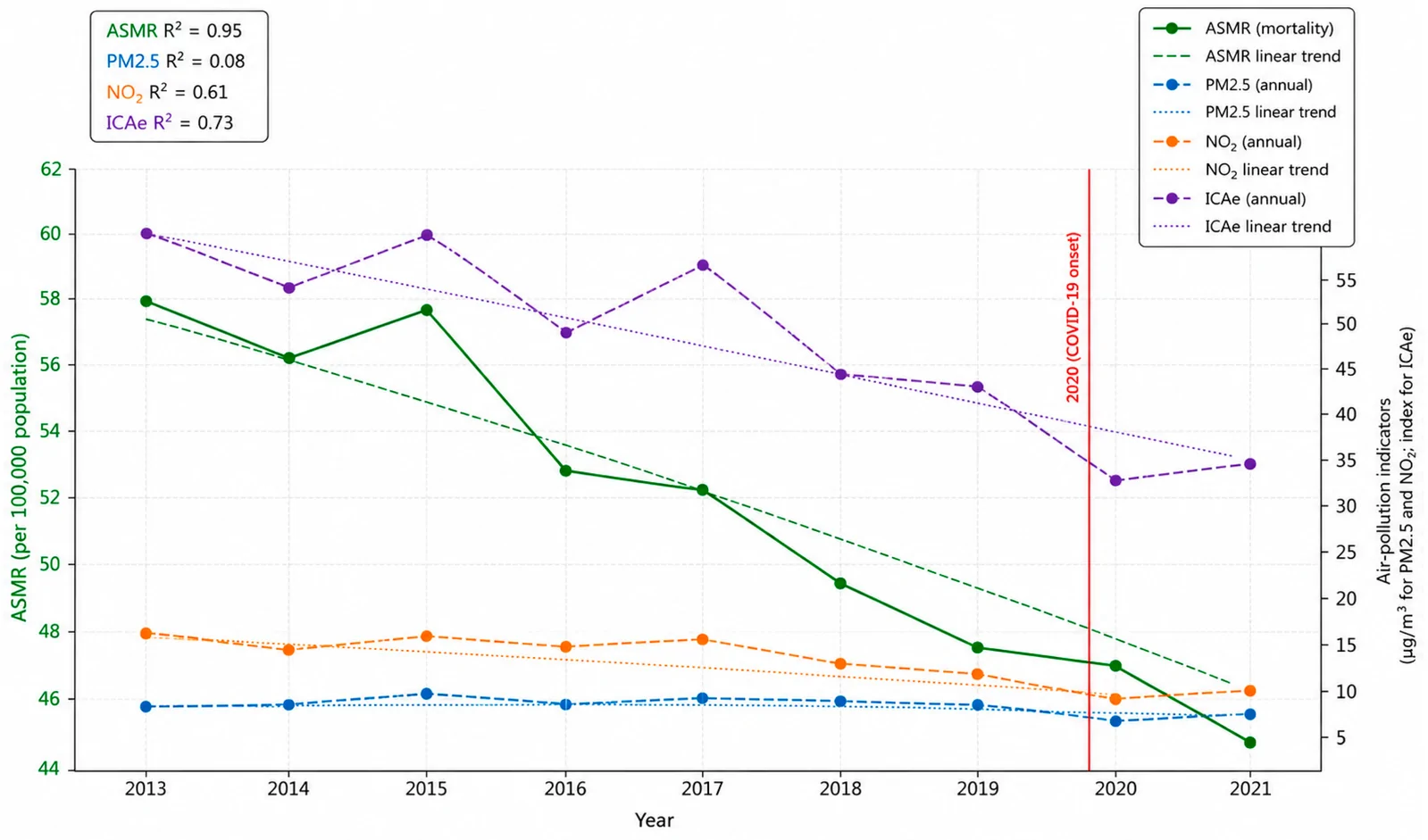
Descriptive Temporal Trends in Age-Standardized Cerebrovascular Mortality and Selected Regional Air-Pollution Measures, 2013–2021. Annual trends in the age-standardized cerebrovascular mortality rate (ASMR), PM2.5, NO_2_, and the composite air quality index (ICAe) from 2013 to 2021. ASMR is shown on the left axis, whereas PM2.5, NO_2_, and ICAe are shown on the right axis. The solid green line represents the annual ASMR values, while the dashed colored lines represent the annual values of PM2.5, NO_2_, and ICAe. The superimposed trend lines represent the corresponding linear temporal trends, with the associated R^2^ values shown in the upper-left corner. The vertical red line marks 2020, corresponding to the onset of the COVID-19 pandemic.

Descriptive temporal trends in pollutants and health outcomes are summarized in Table 4 and visualized in Figure 4. The ASHR decreased significantly (β = −0.321 per year, R^2^ = 0.023, *p* = 0.007), representing an approximately 14.3% reduction from 2013 to 2021. The ASMR showed steepest decline (β = −1.672, R^2^ = 0.146, *p* < 0.001), corresponding to 27.0% decrease. CFR increased modestly (β = 0.266 per year, R^2^ = 0.019, *p* = 0.015). Calendar year explained only a small proportion of the variability in ASHR and IMI.

Benzene declined substantially (β = −0.061 μg/m^3^/year, 47.2% total, *p* < 0.001). NO_2_ (β = −0.515, 30.9% decline, *p* < 0.001), ozone (β = −0.707, 44.9% decline, *p* < 0.001), and ICAe (β = −1.200, 21.1% improvement, *p* < 0.001) also decreased significantly. PM2.5 and PM10 showed non-significant linear trends and exhibited substantial interannual variability.

In 2020, ASHR decreased to 18.0, compared with 19.8 in 2019, whereas CFR increased to 31.2%, compared with 28.7% in 2019. These coincident changes occurred during the first year of the COVID-19 pandemic but should be interpreted cautiously, as the present study did not evaluate pandemic-related changes in healthcare access, admission patterns, or case severity.

### 3.5. Regional Variations

Murcia exhibited the highest age-standardized hospitalization rate (24.3 ± 3.2 per 10,000 population), followed by Asturias (23.2 ± 4.1) and the Valencian Community (23.0 ± 3.8). The Balearic Islands showed the lowest ASHR (15.7 ± 2.9), followed by Castilla-La Mancha (16.8 ± 3.1) and the Canary Islands (18.8 ± 3.5). Catalonia and Madrid had the highest mean NO_2_ concentrations (22.4 and 28.7 μg/m^3^, respectively), whereas Extremadura had the lowest (6.3 μg/m^3^). PM2.5 concentrations were highest in Andalusia and Catalonia (approximately 12.9 μg/m^3^) and lowest in Extremadura and the Balearic Islands (approximately 7.7–8.1 μg/m^3^).

Regions with the highest hospitalization rates did not consistently show the highest pollutant concentrations. Murcia had a high ASHR but moderate PM2.5 concentrations, whereas Madrid had high NO_2_ concentrations but an ASHR close to the national average. This lack of consistent geographical concordance indicates that the available regional pollution indicators alone did not account for the observed variation in hospitalization rates. Unmeasured demographic, socioeconomic, clinical, and healthcare-related characteristics may also have contributed.

Regional differences in hospitalization and pollutant concentrations are detailed in Table 5.

### 3.6. Multivariable Regression Analysis

To examine exploratory ecological associations between PM2.5 and other pollutants and cerebrovascular outcomes, multiple linear regression models were fitted using the age-standardized hospitalization rate (ASHR) and age-standardized mortality rate (ASMR) as dependent variables. Three models were estimated sequentially: a single-pollutant model, a model adjusted for calendar year, and a multipollutant model including co-pollutants and calendar year. All pollutant variables were standardized by mean-centering and scaling to one standard deviation to allow direct comparison of regression coefficients.

#### 3.6.1. Age-Adjusted Hospitalization Rate (ASHR)

In the single-pollutant model, PM2.5 was positively associated with higher ASHR (β = 0.859, 95% CI: 0.207–1.466, *p* = 0.006), with the model explaining 2.5% of variance (R^2^ = 0.025). This association remained nominally significant after adjusting for temporal trend (β = 0.805, 95% CI: 0.224–1.408, *p* = 0.009; R^2^ = 0.045).

However, in the multipollutant model adjusted for calendar year, the association between PM2.5 and ASHR weakened and was no longer statistically significant (β = 0.693, 95% CI: −0.022–1.492, *p* = 0.089; R^2^ = 0.071). Among the other pollutants, benzene remained independently associated with higher hospitalization rates (β = 0.772, *p* = 0.029), while PM10 and SO_2_ showed borderline significance (*p* < 0.10).

#### 3.6.2. Age-Adjusted Mortality Rate (ASMR)

For ASMR, the single-pollutant model showed a positive association with PM2.5 (β = 2.987, 95% CI 1.974–3.999, *p* < 0.001; R^2^ = 0.070). Adjustment for calendar year increased the model R^2^ to 0.203, with PM2.5 remaining positively associated with ASMR (β = 2.698, 95% CI 2.603–3.841, *p* < 0.001). This increase in model fit indicates that temporal variation accounted for an additional proportion of the variability in ASMR.

The multipollutant model adjusted for calendar year produced a larger PM2.5 coefficient (β = 5.696, 95% CI 4.118–7.037, *p* < 0.001; R^2^ = 0.391). The increase in the coefficient after inclusion of co-pollutants may reflect intercorrelations among pollutants, temporal patterns, or model instability. This result should therefore be interpreted cautiously.

In the same model, NO_2_ showed an inverse association with ASMR (β = −5.620, *p* < 0.001), whereas SO_2_ showed a positive association (β = 1.299, *p* = 0.018). O_3_, PM10, benzene, and Pb were not statistically significant in the multipollutant model. Regression coefficients for all models are presented in Table 6.

Variance inflation factors ranged from 1.12 to 1.80 for the individual pollutants and calendar year, suggesting no severe multicollinearity according to conventional thresholds. ICAe was not included in this assessment because of its exact linear dependence on its component variables. Individual VIF values are reported in Supplementary Table S1.

## 4. Discussion

This nine-year ecological study across Spain’s 17 autonomous communities identified marked regional variation in cerebrovascular hospitalization and mortality but only weak and inconsistent ecological associations with annual regional air-pollution measures. The principal findings were as follows: (1) PM2.5 showed a positive association with age-standardized cerebrovascular mortality, although the overall correlation was weak and the coefficient varied across model specifications; (2) inverse correlations involving SO_2_, O_3_, PM10, and ICAe should not be interpreted as protective effects and likely reflect regional pollutant mixtures, exposure misclassification, ecological bias, and residual confounding; (3) substantial regional differences in hospitalization rates were not consistently accompanied by corresponding differences in pollutant concentrations; (4) several pollutants and cerebrovascular mortality declined over time, although these parallel trends do not establish a causal relationship; and (5) sex-stratified correlations showed broadly similar patterns in men and women, but these analyses were descriptive and exploratory because both groups shared the same community-year exposure estimates. Overall, the available annual regional pollution indicators explained only a limited proportion of the geographical variation in cerebrovascular outcomes.

### 4.1. PM2.5 and Cerebrovascular Risk

The positive ecological association between PM2.5 and age-standardized cerebrovascular mortality observed in this study was directionally consistent with previous epidemiological evidence, although its magnitude and interpretation require caution. A systematic review and meta-analysis involving more than 23 million participants reported modest associations between PM2.5 exposure and stroke incidence and mortality [4]. In the ESCAPE project, a 5 μg/m^3^ increase in long-term PM2.5 exposure yielded a hazard ratio of 1.19 for incident cerebrovascular events; however, the wide confidence interval included the null value, indicating that this association was not statistically significant [5].

Direct comparison with these studies is limited by differences in design and exposure assessment. In our analysis, the overall correlation between PM2.5 and ASMR was weak (r = 0.264), and the regression coefficient varied across model specifications. Although variance inflation factors did not indicate severe multicollinearity, correlations among pollutants and temporal patterns may still have influenced the multipollutant estimates. Regional exposure averaging, ecological bias, exposure misclassification, and residual confounding further limit pollutant-specific interpretation. Accordingly, this result should be regarded as an exploratory regional association rather than an estimate of individual exposure-related risk.

European studies using higher-resolution exposure assessments provide complementary evidence. In the population-based ISSYS study in Barcelona, modelled individual exposure to air pollution was associated with covert cerebrovascular abnormalities detected by magnetic resonance imaging [9]. In Poland, the EP-PARTICLES study combined individual clinical outcomes with municipality-specific exposure models and reported associations between short-term PM2.5 exposure and ischemic stroke incidence [6]. A subsequent analysis using high spatial and temporal resolution found an association between short-term PM2.5 exposure and ischemic stroke treated with intravenous thrombolysis [10]. These studies assessed acute exposure and individual clinical events and are therefore not directly comparable with the annual autonomous-community averages used here; nevertheless, they illustrate the additional information provided by finer spatial and temporal resolution.

A Nordic study also documented persistent socioeconomic and geographical inequalities in PM2.5 and NO_2_ exposure despite relatively low average pollution levels [11]. Although cerebrovascular outcomes were not examined, this finding demonstrates that national and regional averages may conceal relevant differences in population exposure. Evidence from China provides a complementary population-level perspective. National clean-air actions were associated with substantial reductions in PM2.5 concentrations between 2013 and 2020 [12], while Global Burden of Disease estimates indicated a decline in the burden of ischemic stroke attributable to air pollution between 1990 and 2021 [13]. Health-impact modelling also estimated mortality benefits attributable to improvements in PM2.5 concentrations between 2014 and 2018 and projected further potential benefits by 2030 [14]. These findings support the potential public-health value of reducing population exposure, but they do not establish that changes in cerebrovascular outcomes were exclusively caused by air-quality interventions.

PM2.5 was more consistently associated with ASMR than with ASHR in the present study. However, this difference should not be interpreted as evidence of a preferential effect on fatal rather than non-fatal cerebrovascular events. Hospitalization and mortality indicators were obtained from different information systems and had different denominators, and data on stroke subtype, clinical severity, prehospital mortality, personal exposure, and individual vascular risk were unavailable. The contrasting results may therefore reflect differences in outcome construction, temporal trends, exposure measurement, or residual confounding rather than a specific effect on stroke lethality.

### 4.2. Interpreting Inverse Associations

The weak inverse correlations observed for SO_2_, O_3_, PM10, and the composite air-quality index (ICAe) should not be interpreted as protective effects. In ecological analyses based on aggregated regional data, inverse associations may arise from ecological bias, residual confounding, exposure misclassification, differences in pollutant sources, urban–rural gradients, regional demographic structure, healthcare access, or correlations within pollutant mixtures [15,16]. Furthermore, because ICAe was derived from its component pollutants, its association should not be interpreted as an independent pollutant effect.

Ozone may show inverse associations with stroke indicators because its spatial distribution often differs from that of traffic-related pollutants such as NO_2_ and benzene. In Europe, higher ozone concentrations are frequently observed in rural or background areas compared with urban traffic environments, partly because of atmospheric transport of ozone and precursors and lower titration by nitrogen oxides away from emission sources [17,18]. Thus, higher regional O_3_ levels may reflect lower local traffic-related pollution rather than lower cerebrovascular risk.

Similarly, PM10 may include a substantial coarse-particle fraction from natural or resuspended sources, including Saharan dust intrusions, which are particularly relevant in Southern Europe and Mediterranean regions. The chemical composition, particle size distribution, and toxicological profile of mineral dust and coarse particles may differ from combustion-related PM2.5, and evidence on the health effects of Saharan dust-related PM10 remains heterogeneous [19,20]. The annual regional averages used in this study did not allow the contribution of different particle sources to be distinguished. SO_2_ may also reflect historical or industrial emission patterns that do not necessarily coincide with current regional cerebrovascular risk profiles.

Therefore, these inverse associations should be interpreted as indicators of complex regional pollutant-mixture patterns rather than evidence of beneficial effects. Future studies using high-resolution exposure assessment, pollutant-mixture methods, and individual-level adjustment for vascular, socioeconomic, and healthcare determinants are needed to clarify these relationships [21].

### 4.3. Regional Heterogeneity and Sex-Stratified Associations

Stroke hospitalization varied approximately 1.5-fold across regions, with potential contributions from geographical, climatic, demographic, socioeconomic, and healthcare-related differences. This regional variation was not consistently aligned with pollutant concentrations, indicating that the annual regional air-pollution measures alone did not adequately explain the geographical differences in hospitalization. Marked geographical differences in stroke incidence, mortality, and disability-adjusted life years have also been documented across European countries [22].

Evidence from the Iberian Peninsula remains limited and methodologically heterogeneous. In Lisbon, Cruz et al. reported associations between several air pollutants and circulatory hospital admissions, including an approximately 1% increase in stroke admissions among adults aged >64 years per 10 μg/m^3^ increase in NO_2_ [23]. At the national level, a Portuguese ecological modelling study estimated a decline in NO_2_-attributable years of life lost from stroke between 2001 and 2021, although it applied an effect estimate derived from the literature rather than estimating a local exposure–outcome association [24]. These studies provide relevant evidence from a neighbouring Mediterranean setting, but differences in exposure windows, geographical resolution, and analytical design limit direct comparison with our repeated regional analysis.

Sex-stratified analyses showed numerically stronger associations among women. However, because men and women shared the same community-year exposure estimates, no formal between-sex comparisons were performed. Therefore, these findings should be interpreted as descriptive and hypothesis-generating rather than as evidence of confirmed female susceptibility. Previous evidence regarding sex-related modification of air-pollution effects is heterogeneous. A meta-analysis of cohort studies found similar PM_2.5_-associated stroke risks in women and men, with no significant women-to-men difference in relative risk [25]. Potential biological mechanisms that might contribute to sex-related heterogeneity are discussed in Section 4.5. Nevertheless, the present ecological analysis cannot establish sex-specific biological vulnerability, and confirmation in individual-level studies with detailed exposure assessment and adjustment for vascular, socioeconomic, and behavioral factors is required.

### 4.4. Clinical and Public Health Implications

The approximately 1.5-fold variation in stroke hospitalization rates across regions, together with the weak and inconsistent ecological associations with PM2.5 and other pollutant indicators, underscores the importance of region-specific assessment of stroke prevention and management strategies. Regions with high stroke burden, such as Murcia, Asturias, and the Valencian Community, warrant further investigation to determine the relative contribution of demographic characteristics, vascular risk factors, healthcare organization, coding practices, and environmental conditions. The present findings alone do not demonstrate that differences in air pollution or stroke-service provision explain their higher hospitalization rates.

The exploratory sex-stratified findings do not provide sufficient evidence to support sex-specific clinical or public health recommendations.

For healthcare systems, these findings suggest the potential value of incorporating environmental indicators into regional stroke surveillance, alongside demographic, socioeconomic, vascular-risk, meteorological, and healthcare-related information. However, environmental data should not be used in isolation for resource allocation because the available annual regional measures explained only a limited proportion of the geographical variation in cerebrovascular outcomes. Continued efforts to reduce population exposure to ambient air pollution remain justified by the broader evidence on its adverse health effects and by current European public health recommendations [26,27]. Future stroke registries and surveillance systems could link clinical information with higher-resolution environmental exposure data to support more informative epidemiological analyses.

### 4.5. Biological Mechanisms

PM2.5 may affect cardiovascular and cerebrovascular health through interacting pulmonary, immune, vascular, autonomic, and hemostatic pathways. Particle deposition in the respiratory tract can generate reactive oxygen species and activate epithelial cells and alveolar macrophages, initiating local inflammation and the systemic release of cytokines and acute-phase mediators. Experimental evidence indicates that PM2.5 uptake by macrophages can activate the NLRP3 inflammasome and promote IL-1β production, providing a direct link between particle exposure and innate immune activation. These inflammatory and oxidative responses may reduce endothelial nitric-oxide bioavailability, impair vascular function, accelerate atherosclerosis and plaque instability, and shift platelet activation, coagulation, and fibrinolysis toward a prothrombotic state. Pulmonary sensory-receptor activation and systemic inflammation may also alter autonomic and neuroendocrine regulation, increasing sympathetic activity, blood-pressure variability, and arrhythmogenic susceptibility [28,29].

At the cerebral level, experimental models suggest that PM2.5 can aggravate ischemia-related injury within the neurovascular unit. Proposed mechanisms include perivascular macrophage activation, loss of endothelial tight-junction proteins, disruption of Akt/β-catenin signalling, and dysregulation of autophagy [30]. The rapid associations between hourly pollutant exposure and emergency stroke admissions reported in multicentre case-crossover studies are compatible with acute triggering pathways, although they do not establish a specific biological mechanism [31]. Heterogeneity across pollutants and stroke subtypes has also been reported, including associations of transient gaseous-pollutant exposure with intracerebral but not subarachnoid haemorrhage [32], suggesting that air pollution is unlikely to act through a single uniform pathway.

Biological sex could modify some of these responses through differences in hormonal and menopausal status, immune regulation, antioxidant capacity, endothelial function, coagulation, and baseline cardiometabolic vulnerability. However, epidemiological evidence remains heterogeneous [25]. Accordingly, the numerical sex-stratified differences observed in the present study should be considered hypothesis-generating rather than evidence of biological interaction. Moreover, biomarkers, personal exposure, stroke subtype, menopausal status, and individual vascular risk factors were not available; therefore, the mechanisms described above provide biological plausibility from external evidence but cannot be inferred directly from this ecological analysis.

### 4.6. Strengths and Limitations

The main strengths of this study are its nationwide coverage of Spain’s 17 autonomous communities, the inclusion of repeated regional observations over nine years, and the use of official health and air-quality data. Age-standardized hospitalization and mortality indicators facilitated comparisons across populations with different age structures, while the simultaneous assessment of several pollutants provided a broad description of regional environmental and cerebrovascular patterns.

Several limitations must be considered. First, the ecological design precludes individual-level and causal inference. Information on vascular risk factors, medication use, socioeconomic conditions, occupational exposures, meteorological factors, and healthcare characteristics was unavailable. These factors may vary across regions and be associated with both pollution and cerebrovascular outcomes; therefore, residual confounding and ecological fallacy remain important concerns.

Second, the 306 sex-specific outcome observations corresponded to 153 unique community-year exposure units because men and women within each community-year shared the same pollutant estimates. Observations from the same autonomous community were also repeated over time. The descriptive correlation, regional-comparison, and temporal-trend analyses did not fully account for paired sex-specific outcomes, within-community dependence, or serial correlation. Consequently, statistical uncertainty may have been underestimated, and sex-stratified and temporal findings should be regarded as exploratory.

Third, autonomous-community annual averages cannot represent personal exposure and may conceal substantial urban–rural and within-region variability. Exposure misclassification is particularly relevant for spatially heterogeneous pollutants such as NO_2_ and benzene and may also arise from differences in monitoring-station distribution, residential mobility, occupational or indoor exposure, and individual activity patterns. Annual averages additionally fail to capture short-term pollution peaks potentially associated with acute cerebrovascular events.

Fourth, the health outcomes were aggregated, pre-calculated administrative indicators, and individual clinical records were unavailable. The 2016 transition from ICD-9-CM to ICD-10-ES and to a new hospital-discharge data model, the progressive inclusion of private hospitals, and regional differences in coding, admissions, transfers, and discharge practices may have affected comparability. The relatively high CFR values may partly reflect the administrative definition, hospitalized case mix and severity, and regional admission or transfer practices; they should not be interpreted as population-based 30-day case fatality. Moreover, the data did not distinguish ischemic from hemorrhagic stroke or include severity, treatment, functional outcome, or prehospital mortality. Differences between hospitalization and mortality indicators may therefore reflect their distinct definitions and data sources rather than a preferential association with fatal events.

Fifth, the study period included the COVID-19 pandemic. Changes in healthcare-seeking behaviour, emergency access, admission thresholds, hospital organization, coding, case severity, and competing mortality during 2020–2021 may have influenced hospitalization and hospital-outcome indicators. The lower ASHR and higher CFR observed in 2020 coincided with the onset of the pandemic, but their causes cannot be determined from the available data. Because no pandemic-specific sensitivity analysis was performed, its influence on temporal and regression estimates cannot be excluded.

Finally, numerous pollutant–outcome associations were examined. Most nominal findings did not meet the Bonferroni-corrected threshold, overall correlations were weak and inconsistent, and some coefficients varied across model specifications. Although VIF values did not indicate severe multicollinearity, correlations among pollutants may still have influenced multipollutant estimates. The findings should therefore be interpreted as exploratory population-level signals of limited explanatory value rather than as pollutant-specific or individual risk estimates.

Despite these limitations, the ecological design remains useful for describing regional disparities and broad temporal patterns and for evaluating whether routinely available pollution indicators correspond with geographical variation in cerebrovascular outcomes.

### 4.7. Policy Implications and Future Research Directions

Taken together, these findings support prioritizing PM2.5 reduction efforts to meet the WHO’s recommended threshold of ≤5 μg/m^3^, a target substantially stricter than current EU limits [1,26]. According to modeling estimates, achieving these levels could prevent thousands of premature deaths each year [33,34]. Because regional heterogeneity was evident, targeted interventions appear warranted—particularly in urban regions, where baseline pollution burden tends to be higher [22]. Harmonizing exposure limits and reducing health inequities will also require closer coordination with broader European policy frameworks [31]. Notably, improvements in air quality carry an added advantage: they align with climate mitigation goals, reinforcing the case for co-benefit policy frameworks [2].

Looking ahead, future research should move toward individual-level cohort studies that incorporate high-resolution exposure assessment, stroke subtype analysis, and biomarkers of inflammation and endothelial injury. Acute exposure effects, meanwhile, could be clarified through time-series and case-crossover designs, while interaction analyses may help identify particularly vulnerable subgroups. Finally, economic evaluations that quantify the health-system benefits of pollution mitigation would strengthen the case for policy prioritization [35].

## 5. Conclusions

This nationwide ecological study identified marked regional disparities in cerebrovascular hospitalization and mortality across Spain’s autonomous communities from 2013 to 2021. PM2.5 showed a positive ecological association with age-standardized cerebrovascular mortality, although the overall correlation was weak and the regression coefficient varied across model specifications. Its association with hospitalization was weaker and was no longer statistically significant after adjustment for co-pollutants and calendar year.

Sex-stratified findings were exploratory and did not provide evidence of sex-specific susceptibility. Several inverse pollutant–outcome associations should not be interpreted as protective effects and may reflect residual confounding, or regional pollutant-mixture and contextual patterns.

Overall, annual regional air-pollution indicators explained only a limited proportion of the geographical variation in cerebrovascular outcomes. These findings highlight the need to investigate demographic, vascular, socioeconomic, environmental, and healthcare-related determinants of regional disparities.

## Figures and Tables

**Table 1:** Descriptive statistics of cerebrovascular health indicators and annual regional air-pollution measures, Spain, 2013–2021.

Variable	Mean ± SD	Median (IQR)	Range
Health indicators			
CHR	21.4 ± 4.6	21.2 (18.2–24.2)	5.6–34.4
ASHR	20.1 ± 5.4	19.0 (15.4–24.6)	5.9–33.9
ASMR	51.8 ± 11.3	50.8 (43.7–59.3)	27.5–86.5
CMR	11.2 ± 4.0	10.2 (7.7–14.4)	4.5–22.8
IMI	10.9 ± 2.9	10.5 (8.5–13.3)	5.4–19.6
CFR	29.5 ± 5.0	29.2 (25.6–32.8)	19.0–47.2
Air-pollution indicators			
PM2.5 (µg/m^3^)	9.96 ± 1.88	9.70 (8.68–11.00)	6.57–15.52
PM10 (µg/m^3^)	19.3 ± 4.2	18.4 (16.0–22.3)	11.8–37.7
NO_2_ (µg/m^3^)	15.0 ± 5.7	13.9 (10.8–18.0)	4.9–34.2
SO_2_ (µg/m^3^)	2.27 ± 1.94	1.73 (0.91–3.06)	0.00–10.00
O_3_ (µg/m^3^)	11.5 ± 8.6	9.86 (4.63–16.93)	0.09–35.70
CO (µg/m^3^)	1.22 ± 0.49	1.14 (0.87–1.49)	0.43–3.30
C_6_H_6_, benzene (µg/m^3^)	0.66 ± 0.50	0.50 (0.32–0.86)	0.01–2.70
Pb, lead (µg/m^3^)	0.012 ± 0.020	0.005 (0.002–0.012)	0.004–0.110
ICAe	48.0 ± 13.5	44.0 (38.5–53.8)	27.3–89.9

CHR (Crude hospitalization rate, per 10,000 population); ASHR (Age-standardized hospitalization rate, per 10,000 population); ASMR (Age-standardized cerebrovascular mortality rate, per 100,000 population); CMR (Age-standardized premature cerebrovascular mortality rate among persons aged <75 years, per 100,000 population); IMI (In-hospital mortality index); CFR (In-hospital case fatality, %); ICAe, composite air quality index; IQR, interquartile range; SD, standard deviation.

**Table 2:** Selected Correlations Between Annual Regional Air-Pollution Measures and Cerebrovascular Health Indicators.

Health Indicator	Pollutant	Pearson r	95% CI	*p*-Value
ASMR	PM2.5	0.264	0.153, 0.369	<0.001 ***
CHR	SO_2_	−0.235	−0.345, −0.120	<0.001 ***
CFR	ICAe	−0.234	−0.344, −0.119	<0.001 ***
CHR	O_3_	−0.232	−0.342, −0.117	<0.001 ***
CHR	ICAe	−0.229	−0.339, −0.114	<0.001 ***
CFR	NO_2_	−0.210	−0.321, −0.094	<0.001 ***
ASMR	SO_2_	0.200	0.086, 0.310	<0.001 ***
CHR	PM10	−0.198	−0.308, −0.083	<0.001 ***
CHR	Pb	0.192	0.077, 0.303	0.001 **
ASHR	C_6_H_6_	0.185	0.070, 0.297	0.001 **
ASHR	PM2.5	0.158	0.042, 0.271	0.006 **
CFR	SO_2_	−0.142	−0.255, −0.025	0.013 *
ASHR	NO_2_	0.141	0.025, 0.254	0.013 *
IMI	NO_2_	−0.141	−0.254, −0.024	0.014 *
CMR	PM2.5	0.139	0.023, 0.253	0.015 *

Values shown correspond to the strongest pollutant–health indicator correlations with nominal *p* < 0.05. The Bonferroni-corrected threshold for 54 comparisons was *p* < 0.0009. Associations not meeting this threshold should be interpreted as exploratory. * *p* < 0.05; ** *p* < 0.01; *** *p* < 0.001.

**Table 3:** Exploratory Sex-Stratified Correlations Between Annual Regional Air-Pollution Measures and Hospitalization Indicators (*n* = 153 per sex).

Pollutant	Men r (95% CI)	*p*-Value	Women r (95% CI)	*p*-Value	Δr
ASHR correlations					
C_6_H_6_	0.338 (0.195–0.466)	<0.001	0.364 (0.225–0.488)	<0.001	0.026
PM2.5	0.320 (0.175–0.450)	<0.001	0.296 (0.149–0.429)	<0.001	−0.024
NO_2_	0.260 (0.111–0.397)	0.001	0.308 (0.162–0.439)	<0.001	0.048
CHR correlations					
SO_2_	−0.229 (−0.368, −0.078)	0.004	−0.312 (−0.442, −0.168)	<0.001	−0.083
O_3_	−0.268 (−0.403, −0.121)	0.001	−0.249 (−0.387, −0.099)	0.002	0.019
ICAe	−0.267 (−0.402, −0.120)	0.001	−0.246 (−0.384, −0.095)	0.002	0.021

ASHR, age-standardized hospitalization rate; CHR, crude hospitalization rate; CI, confidence interval; ICAe, composite air quality index. Δr represents the descriptive difference between the correlation coefficient in women and that in men; no formal statistical comparison was performed.

**Table 4:** Descriptive Temporal Trends in Cerebrovascular Health Indicators and Annual Regional Air-Pollution Measures, Spain, 2013–2021.

Variable	β (per Year)	SE	R^2^	*p*-Value	% Change 2013–2021
Health indicators					
ASHR	−0.321	0.115	0.023	0.007 **	−14.3%
ASMR	−1.672	0.244	0.146	<0.001 ***	−27.0%
CMR	−0.141	0.089	0.008	0.116	−11.3%
CFR	0.266	0.108	0.019	0.015 *	+8.1%
Air pollutants measures					
C_6_H_6_	−0.061	0.011	0.099	<0.001 ***	−47.2%
NO_2_	−0.515	0.125	0.054	<0.001 ***	−30.9%
O_3_	−0.707	0.187	0.045	<0.001 ***	−44.9%
ICAe	−1.200	0.295	0.053	<0.001 ***	−21.1%
PM2.5	−0.051	0.042	0.005	0.223	−4.6%
PM10	0.099	0.093	0.004	0.288	+4.6%

β represents the estimated annual change from ordinary least-squares regression with calendar year as the independent variable. For ASHR, β is expressed per 10,000 population per year; for ASMR and CMR, per 100,000 population per year; for CFR, in percentage points per year; for pollutants, in µg/m^3^ per year, except ICAe, which is expressed in index units per year. The percentage change represents the relative change between 2013 and 2021. SE, standard error. * *p* < 0.05; ** *p* < 0.01; *** *p* < 0.001.

**Table 5:** Regional Variations in Age-Standardized Hospitalization Rates and Selected Annual Air- Pollutants Measures, Spain, 2013–2021.

Autonomous Community	ASHR †	PM2.5	PM10	NO_2_
Murcia	24.3	12.4	23.5	19.2
Asturias	23.2	11.0	23.8	17.8
Valencian Community	23.0	9.7	16.2	12.7
Basque Country	21.7	8.9	16.2	19.3
Extremadura	21.1	7.7	14.1	6.3
Catalonia	20.8	12.9	22.4	22.4
La Rioja	20.6	8.7	18.2	9.0
Navarre	20.2	10.3	15.1	13.7
Madrid	20.2	10.4	18.9	28.7
Andalusia	20.0	12.9	24.3	14.9
Cantabria	19.2	9.3	19.2	15.6
Aragon	19.0	10.3	16.7	16.8
Galicia	19.0	10.0	15.5	11.4
Canary Islands	18.8	8.6	26.2	11.8
Castile and León	18.4	8.2	16.1	11.7
Castilla-La Mancha	16.8	9.7	22.9	13.9
Balearic Islands	15.7	8.1	18.2	9.8
National mean	20.1	10.0	19.3	15.0

† ASHR = age-standardized hospitalization rate; PM2.5 = fine particulate matter (μg/m^3^); PM10 = coarse particulate matter (μg/m^3^); NO_2_ = nitrogen dioxide (μg/m^3^). All values represent 2013–2021 regional means.

**Table 6:** Multivariable Regression Coefficients for PM2.5: Univariate, Adjusted, and Multivariable Models.

Outcome	Model	β per 1-SD PM2.5 Increase	95% CI	*p*-Value	R^2^	Adj R^2^
ASHR	Univariate	0.859	0.207–1.466	0.006 **	0.025	0.022
ASHR	PM2.5 + Year	0.805	0.224–1.408	0.009 **	0.045	0.039
ASHR	All pollutants + Year	0.693	−0.022–1.492	0.089	0.071	0.046
ASMR	Univariate	2.987	1.974–3.999	<0.001 ***	0.070	0.067
ASMR	PM2.5 + Year	2.698	2.603–3.841	<0.001 ***	0.203	0.198
ASMR	All pollutants + Year	5.696	4.118–7.037	<0.001 ***	0.391	0.375

** *p* < 0.01; *** *p* < 0.001. β = change in the outcome per one standard deviation increase in PM2.5 concentration. ASHR = age-standardized hospitalization rate; ASMR = age-standardized mortality rate; Adj R^2^ = adjusted coefficient of determination.

## Data Availability

The original contributions presented in this study are included in the article/Supplementary Material. Further inquiries can be directed to the corresponding author(s).

## Supplementary Materials

The following supporting information can be downloaded at: https://www.mdpi.com/article/10.3390/medsci14040475/s1, Table S1, variance inflation factors for predictors included in the multipollutant regression models; Supplementary File S1, completed STROBE checklist.
