# Supplementary material for: Regional Disparities in Stroke Hospitalization and Mortality Associated with Long-Term Fine Particulate Matter Exposure: A Nationwide Ecological Study in Spain
Source: Med Sci (Basel). 2026 Aug 12;14(4):475. doi: 10.3390/medsci14040475 (PMC13515196; doi:10.3390/medsci14040475)
Supplement: Supplementary file 1 [file medsci-14-00475-s001.zip › medsci-4441387-supplementary.pdf]

Supporting Information for Publication

STROBE Checklist

Regional Disparities in Stroke Hospitalization and Mortality Associated with Long-Term Fine Particulate Matter Exposure: A Nationwide Ecological Study in Spain

José María Ramírez-Moreno [1,2,*], Víctor Martín Hernández [3], Andrea Parejo Olivera [1], Noelia Valverde Mata [1], Marina Mesa Hernández [1], Pablo Macías Sedas [1], Ana Maria Roa Montero [1], María José Gómez Baquero [1], David Ceberino Muñoz [1] and David Tena Mora [1]

[1] Stroke Center, Neurology Department, University Hospital of Badajoz, 06080 Badajoz, Extremadura, Spain
[2] Department of Biomedical Sciences, University of Extremadura, 06080 Badajoz, Extremadura, Spain
[3] Pneumology Department, University Hospital of Badajoz, 06080 Badajoz, Extremadura, Spain
* Correspondence: jramrez@unex.es; Tel.: +34-924-218-100 (ext. 48386)

Checklist adaptation note: The STROBE checklist is provided for this observational nationwide longitudinal ecological study. Items referring to individual participant recruitment, follow-up, or exposed/unexposed groups are marked as not applicable when appropriate, because the analysis used aggregated regional data rather than individual-level cohort data.

Item	Section/Topic	STROBE item	Location in manuscript	Status	Notes/adaptation for ecological study
1	Title/Abstract	Indicate the study's design with a commonly used term in the title or the abstract; provide an informative and balanced summary of methods and results.	Title; Abstract	Yes	The title identifies the work as a nationwide ecological study; the abstract summarizes background, objectives, methods, results, and conclusions.
2	Background/rationale	Explain the scientific background and rationale for the investigation being reported.	Introduction	Yes	The Introduction describes stroke burden, regional heterogeneity, PM2.5 as an environmental risk factor, and the limited evidence from Mediterranean Europe.
3	Objectives	State specific objectives, including any prespecified hypotheses.	Introduction	Yes	The objective is to describe regional disparities in cerebrovascular hospitalization and mortality and examine their ecological associations with annual regional air-pollution measures.
4	Study design	Present key elements of study design early in the paper.	Methods: Study Design and Setting	Yes	The study is described as a nationwide longitudinal ecological study using regional-level aggregated data.
5	Setting	Describe the setting, locations, and relevant dates, including periods of exposure, follow-up, and data collection.	Methods: Study Design and Setting; Environmental Data	Yes	The setting includes Spain's 17 autonomous communities during 2013-2021, with annual regional air pollution and stroke outcome data.
6	Participants	Give eligibility criteria, sources, and methods of selection of participants. For matched studies, give matching criteria and numbers of exposed/unexposed.	Methods: Study Design and Setting; Study Population and Variables	N/A/Yes	This is an ecological study without individual participant recruitment. The population included residents of Spain; the analytical units were region-year-sex observations.

Item	Section/Topic	STROBE item	Location in manuscript	Status	Notes/adaptation for ecological study
7	Variables	Clearly define all outcomes, exposures, predictors, potential confounders, and effect modifiers. Give diagnostic criteria if applicable.	Methods: Data Sources; Environmental Data; Study Population and Variables	Yes	Stroke hospitalization indicators were defined using ICD-9-CM codes 430–438 through 2015 and ICD-10-ES codes I60–I69 from 2016. Cerebrovascular mortality indicators were based on ICD-10 codes I60–I69. Pollutants, sex, calendar year, and autonomous community are defined in the Methods
8	Data sources/measurement	For each variable of interest, give sources of data and details of methods of assessment or measurement. Describe comparability of assessment methods if relevant.	Methods: Data Sources; Environmental Data	Yes	Health indicators were retrieved as aggregated, pre-calculated measures from INCLASNS; the underlying sources were RAE-CMBD for hospitalization indicators and INE statistics for mortality indicators. Air-pollution data were obtained from official MITECO datasets. The procedure used to derive annual regional pollutant measures is described in the Methods.
9	Bias	Describe any efforts to address potential sources of bias.	Methods: Study Design and Setting; Statistical Analysis; Discussion: Strengths and Limitations	Yes	The manuscript addresses ecological inference, non-independence of sex-stratified observations, multiple comparisons, residual confounding, exposure misclassification, and ecological fallacy.
10	Study size	Explain how the study size was arrived at.	Methods: Study Design and Setting; Results: Descriptive Statistics	Yes	The dataset included 306 sex-specific community-year outcome observations, corresponding to 153 unique community-year environmental exposure units. No individual participant recruitment occurred.
11	Quantitative variables	Explain how quantitative variables were handled in the analyses. If applicable, describe which groupings were chosen and why.	Methods: Statistical Analysis; Tables 1-6	Yes	Continuous variables were summarized descriptively. Pollutant concentrations were standardized in the regression models, and regional mean values are presented in Table 5.
12	Statistical methods	Describe all statistical methods, including those used to control for confounding; describe methods for subgroups and interactions; explain handling of missing data; describe sensitivity analyses if applicable.	Methods: Statistical Analysis	Yes	The manuscript describes Pearson correlations, Bonferroni correction, descriptive sex-stratified analyses, OLS temporal trends, ANOVA/Tukey tests, multipollutant linear regression, and VIF assessment. No formal between-sex comparison or pandemic-specific sensitivity analysis was performed.
13	Participants/results	Report numbers of individuals at each stage of the study and reasons for non-participation, if applicable. Consider use of a flow diagram.	Results: Descriptive Statistics; Table 1	N/A/Yes	The dataset included 306 sex-specific community-year outcome observations, corresponding to 153 unique community-year environmental exposure units. No individual participant recruitment occurred.
14	Descriptive data	Give characteristics of study participants and information on exposures and potential confounders; indicate number of participants with missing data for each variable; summarize follow-up time if relevant.	Results: Descriptive Statistics; Table 1	Yes	Table 1 summarizes cerebrovascular health indicators and annual regional pollutant concentrations.

Item	Section/Topic	STROBE item	Location in manuscript	Status	Notes/adaptation for ecological study
15	Outcome data	Report numbers of outcome events or summary measures over time.	Results; Tables 1 and 4; Figures 1-4	Yes	Stroke hospitalization, mortality, and in-hospital mortality indicators are summarized in tables and temporal trends are presented.
16	Main results	Give unadjusted and, if applicable, confounder-adjusted estimates and their precision. Make clear which confounders were adjusted for and why included.	Results: Correlation Analysis; Multivariate Regression Analysis; Tables 2 and 6	Yes	The manuscript reports correlations with confidence intervals and multivariable regression coefficients adjusted for year and co-pollutants.
17	Other analyses	Report other analyses done, such as analyses of subgroups and interactions, and sensitivity analyses.	Results: Sex-Stratified Analysis; Temporal Trends; Regional Variations	Yes	Sex-stratified correlations, temporal trends, and regional comparisons are reported. Sex-stratified analyses were considered descriptive and exploratory; no formal between-sex test was performed.
18	Key results	Summarize key results with reference to study objectives.	Discussion	Yes	The Discussion summarizes PM2.5-ASMR associations, inverse correlations, regional variation, temporal changes, and exploratory sex-stratified findings.
19	Limitations	Discuss limitations, taking into account sources of potential bias or imprecision. Discuss both direction and magnitude of any potential bias.	Discussion: Strengths and Limitations	Yes	The limitations section addresses ecological inference, residual confounding, exposure misclassification, shared sex-specific exposure estimates, repeated regional observations, coding changes, the COVID-19 period, lack of stroke-subtype information, and weak and inconsistent effect estimates.
20	Interpretation	Give a cautious overall interpretation of results considering objectives, limitations, multiplicity of analyses, results from similar studies, and other relevant evidence.	Discussion; Conclusions	Yes	The manuscript interprets results as ecological associations and hypothesis-generating findings, with explicit caution regarding multiple testing and ecological inference.
21	Generalisability	Discuss the generalisability or external validity of the study results.	Discussion: Strengths and Limitations; Public Health Implications	Yes	The manuscript discusses relevance to Mediterranean European regions and the need for individual-level studies before generalizing to individual risk.
22	Funding	Give the source of funding and the role of funders for the present study and, if applicable, for the original study on which the article is based.	Title page: Funding	Yes	The manuscript states that no specific funding was received.

Abbreviations: ASHR, age-standardized hospitalization rate; ASMR, age-standardized mortality rate; CFR, in-hospital case fatality rate; CHR, crude hospitalization rate; CMR, age-standardized premature cerebrovascular mortality rate among persons aged <75 years.; IMI, in-hospital mortality index; PM2.5, fine particulate matter; VIF, variance inflation factor.

No tracked changes, comments, highlighting, or review-only content are included in this Supporting Information for Publication file.

Supplementary Table S1. Variance Inflation Factors for Predictors Included in the Multipollutant Regression Models

Predictor	VIF
$PM_{2.5}$	1.80
PM_{10}	1.66
NO_2	1.72
SO_2	1.14
O_3	1.33
CO	1.18
C_6H_6	1.33
Pb	1.12
Calendar year	1.23

VIF, variance inflation factor. VIFs were calculated for the predictors included in the multipollutant regression models. The same values apply to the ASHR and ASMR models because both included the same set of predictors. ICAe was not entered into the multipollutant models because it was derived from its component pollutants and was therefore not considered an independent predictor.
